# Prognosis in Hispanic patient population with pulmonary arterial hypertension: An application of common risk stratification models

**DOI:** 10.1002/pul2.12209

**Published:** 2023-04-01

**Authors:** Kahtan Fadah, Jose B. Cruz Rodriguez, Haider Alkhateeb, Debabrata Mukherjee, Hernando Garcia, Dan Schuller, Khan O. Mohammad, Sandeep Sahay, Nils P. Nickel

**Affiliations:** ^1^ Department of Internal Medicine Texas Tech University Health Sciences Center El Paso El Paso Texas USA; ^2^ Department of Internal Medicine, Division of Cardiovascular Medicine University of California San Diego San Diego California USA; ^3^ Department of Internal Medicine, Texas Tech University Health Sciences Center El Paso Division of Cardiovascular Medicine El Paso Texas USA; ^4^ Department of Internal Medicine, Division of Pulmonology and Critical Care Medicine Mount Sinai Medical Center Miami Beach Florida USA; ^5^ Department of Internal Medicine Texas Tech University Health Science Center El Paso‐ Transmountain El Paso Texas USA; ^6^ Department of Internal Medicine, Dell Seton Medical Center University of Texas at Austin Austin Texas USA; ^7^ Houston Methodist Hospital Lung Center Houston Methodist Hospital Houston Texas USA; ^8^ Department of Internal Medicine, Division of Pulmonary and Critical Care Texas Tech University Health Sciences Center El Paso El Paso Texas USA

**Keywords:** COMPERA, Hispanic population, PAH prognostic tools, pulmonary arterial hypertension, REVEAL

## Abstract

Pulmonary arterial hypertension (PAH) is a cardiovascular disease with high mortality rate. Current guidelines propose initiation and escalation of PAH‐targeted treatment based on a goal‐directed approach targeting hemodynamic, functional, and biochemical variables. This approach has been successfully validated in large Caucasian cohorts. However, given the low number of Hispanic patients enrolled in large PAH trials and registries, it is unknown if the same prognostic tools can be applied to this patient population. We analyzed a single‐center outpatient cohort that consisted of 135 Hispanic patients diagnosed with PAH. Baseline characteristics were calculated based on COMPERA, COMPERA 2.0 and REVEAL 2.0 risk scores before the initiation of PAH‐targeted therapies. The survival rate at 1 year after diagnosis was 88% for the entire cohort. The three established risk scores to predict PAH outcomes yielded similar results with reasonable discrimination of mortality in the different risk strata (all *p* < 0.001). Hispanic patients with PAH have a high mortality rate. Our analysis suggests that guideline proposed risk assessment at baseline yields important prognostic information in this patient population.

## INTRODUCTION

Pulmonary arterial hypertension (PAH) is a progressive disease of the pulmonary vasculature and an important cause of right heart failure and death. Over the years, treatment options for PAH have evolved, leading to substantial improvement in quality of life and outcomes.^1^, ^2^, ^3^, ^4^ Currently, several PAH‐targeted therapies have been approved to treat PAH and the list is expanding. Present PAH‐targeted therapies range from single‐drug therapy to triple combination therapies and are selected based on a multiparametric risk assessment and individual patient prognostication. Many of these risk assessments in PAH patients are the sum of several nonmodifiable risk factors such as age, gender, type of PAH and modifiable factors such as comorbidities and symptoms.^5^, ^6^, ^7^, ^8^ Large national and international PAH patient registries have been used to derive and validate clinically relevant variables associated with survival, yielding accurate discrimination of mortality at baseline and follow‐up. Current European and American guidelines propose multidimensional, dynamic risk stratification based on functional status, exercise capacity, hemodynamics, echocardiography, blood biomarkers and pulmonary function testing.^9^, ^10^, ^11^, ^12^

Most of these risk assessment tools are derived from PAH registries, which are mainly comprised of a Caucasian population and only a small percentage of patients were of Hispanic ethnicity. For example, in the REVEAL registry, less than 10% of the enrolled patients were identified as Hispanics.^13^ Very little is known about the Hispanic PAH population with regard to risk assessment, outcomes and response to therapy. In a precision medicine approach, recent cardiovascular disease (CVD) guidelines for heart failure (HF) and systemic hypertension (HTN) have addressed therapies in different ethnical and vulnerable populations with specific recommendations.^14^, ^15^ Presently, no proposed ethnicity‐specific management for PAH patients exist. Therefore, it is unknown whether the currently used algorithms to estimate survival apply to different PAH ethnicities at baseline or after initiation of PAH‐targeted therapies. This study aimed to investigate if commonly used risk stratification strategies for PAH carry similar prognostic values in Hispanic patients at baseline. Hence, we analyzed data from a single‐center Hispanic PAH cohort to validate commonly used prognostication tools (such as the REVEAL score and a modified version of the ESC/ERS 3‐ and 4‐strata models for PAH).

## METHODS

### Study population

This was a single‐center retrospective study conducted in a large referral center, University Medical Center (UMC) at the American Mexican border with access to all advanced PAH‐targeted therapies. Of 232 total patients with PAH, 135 Hispanic PAH patients who met the inclusion criteria and were consecutively managed at the University Center between January 2015 and December 2021 were included. Patients were enrolled in this registry as inpatients with outpatient follow‐up or as outpatients only. Since the hemodynamic definition of PAH has changed, we used different enrollment criteria before and after 2019. We chose only patients who met the following criteria based on previous and most recent guidelines^10^: (1) treatment‐naïve with newly diagnosed PAH, (2) initiation of PAH‐targeted therapies within 6 months after diagnosis, (3) mean pulmonary artery pressure >25 mmHg (>20 mmHg after 2019), pulmonary artery wedge pressure <15 and pulmonary vascular resistance (PVR) >3 wood units (PVR > 2 after 2019), (4) exclusion of WHO‐group 2, 3, and 4 PH based on past medical history, echocardiography, high‐resolution chest imaging, pulmonary function testing, and ventilation perfusion scans. PAH‐targeted therapies were reported within 3 months after diagnosis of PAH. Ethnicity was self‐reported and extracted from patients' medical records.

### Follow‐up and outcome definitions

All patients were followed‐up with regular outpatient visits after the diagnosis of PAH. Survival was assessed from the date of the initial diagnosis and as a composite endpoint of death or lung transplantation. The survival status was censored in January 2021. All outcomes were retrospectively extracted from medical records. Mortality was based on reviewing the UMC medical record documentation such as discharge summaries.

### Study measures

Risk stratification based on European Society of Cardiology (ESC) and European Respiratory Society (ERS) Guidelines:

We categorized patients as low, intermediate, intermediate low, intermediate high, or high risk based on two abbreviated versions of the 2022 European PAH guidelines, including the World Health Organization Functional Class (WHO‐FC), 6‐min walking distance (6mwd), NT‐pro‐B‐type Natriuretic Peptide (NT‐proBNP).^9^, ^10^ Each variable was graded from 1 to 3 or from 1 to 4, and the mean was calculated by dividing the sum of all grades by the number of variables and rounded to the next integer (see Supporting Information).

Risk stratification based on Registry to Evaluate Early and Long‐Term PAH Disease Management (REVEAL 2.0):

Patients were classified into low‐, intermediate‐, and high‐risk groups based on the REVEAL 2.0 scoring system (see Supporting Information).

### Statistical analysis

All variables were assessed for symmetrical distributions. Means and standard deviations were reported for variables with uniform distribution. For asymmetrical variables, the median and interquartile ranges were reported. Table 1 displays data as absolute numbers or percentages. In Table 2, the data are shown as absolute numbers with percentages. Kaplan–Meier graphs were used to estimate survival during follow‐up based on REVEAL 2.0 or ESC risk scores. The log‐rank test was used to assess the estimated survival between different risk categories. Receiver operating characteristics curves and corresponding c‐statistics to compare discrimination between the risk scores were calculated for REVEAL 2.0, COMPERA 1.0 and 2.0. All analyses were performed using SPSS 29 and GraphPad Prism 8.0.

**Table 1:** Baseline hemodynamic treatment characteristics, values, medical comorbidities, vital signs, and hospitalization rates.

Variables	All patients baseline (*n* = 135)
Median age (years) (IQR)	57 (15)
Female [%]	76
BMI	30.2 (12)
*Medical comorbidities*	
Obesity (BMI > 30)	41.2%
Systemic hypertension	44.0%
COPD	8.6%
Diabetes mellitus	11.3%
Chronic kidney disease	21.5%
Smoking	7.1%
WHO‐FC	56, 65, 14
*PAH*	
IPAH	68 (50.4%)
CTD‐PAH	33 (24.4%)
CHD‐PAH	28 (20.8%)
PoPH	3 (2.2%)
D&T‐PAH	3 (2.2%)
mRAP [mmHg]	9.2 (5.9)
mPAP [mmHg]	56 (26)
PCWP [mmHg]	9 (3)
CI [l/min/m2]	2.3 (0.6)
SvO2	64(8)
6mwd [meter]	349 (118)
NTproBNP [ng/l]	1932 (3383)
CKD	21.5%
SBP	109 (15)
HR	85 (17)
All‐cause hospitalizations (<6 month)	6%
Pericardial effusion	5%
% Predicted DLCO < 40%	12%
RAP > 20 mmHg (within 1 year)	8%
PVR < 5 Wood units	21%

*Note*: Median values with interquartile range (IQR) were used in the applicable data points. Data are presented in percentage %.

Abbreviations: 6mwd, six‐minute walk test; BMI, body mass index; CHD‐PAH, congenital heart disease‐PAH; CI, cardiac index; CKD, chronic kidney disease; COPD, chronic obstructive pulmonary disease; CTD‐PAH, connective tissue disease‐PAH; DLCO, diffusing capacity for carbon monoxide; D&T‐PAH, toxin‐induced pulmonary arterial hypertension; HR, heart rate; IPAH, idiopathic PAH; mPAP, mean pulmonary artery pressure; mRAP, mean right arterial pressure; NTproBNP, N‐terminal (NT)‐pro hormone B‐type natriuretic peptide; PAH, pulmonary arterial hypertension etiology; PCWP, pulmonary capillary wedge pressure; PoPH, portopulmonary hypertension; PVR, pulmonary vascular resistance; RAP, right atrial pressure; SBP, systolic blood pressure; SvO2, mixed venous oxygen saturation; WHO‐FC, World Health Organization‐functional class.

**Table 2:** Stratification into three categories low‐, intermediate‐and high‐risk based on the three risk assessments tools Registry to Evaluate Early and Long‐term PAH Disease Management (REVEAL 2.0), Comparative, Prospective Registry of Newly Initiated Therapies for Pulmonary (COMPERA 1.0, and COMPERA 2.0).

*N* (%)	REVEAL 2.0	COMPERA		COMPERA 2.0
Low	63 (46.7)	37 (27.4)		24 (18%)
Intermediate	20 (14.8)	76 (56.3)	Low	49 (36%)
			High	47 (35%)
High	50 (37.0)	22 (16.3)		15 (11%)

## RESULTS

The median age of this cohort was 57 years (interquartile range [IQR]) 15, of which 76% were female. The database consists of 135 Hispanic patients diagnosed with PAH from 235 patients with PAH. Half of the patients had idiopathic pulmonary arterial hypertension (IPAH50.4%), followed by connective tissue disease‐associated pulmonary arterial hypertension (CTD‐PAH,24.4%), congenital heart disease‐associated pulmonary arterial hypertension (CHD‐PAH,20.8%), portopulmonary hypertension (PoPH, 2.2%), and drug‐ and toxin‐induced pulmonary arterial hypertension (D&T‐PAH,2.2%). Most patients were classified as WHO‐FC III (65, 48%). In addition, 21% of the entire cohort had chronic kidney disease (CKD) based on the KDIGO criteria, 12% had reduced DLCO on initial pulmonary function testing, and 5% had evidence of pericardial effusion on baseline echocardiography. Baseline hemodynamic characteristics, medical comorbidities, NT‐proBNP values, vital signs, and hospitalization rates are shown in Table 1.

At baseline, only four patients (2.9%) were vasoreactive and treated with calcium channel blocker (CCB) therapy, while 46 patients (34%) and 89 patients (66%) were on PAH‐targeted monotherapy and combination therapy, respectively. The entire cohort's median follow‐up duration was 2.9 years (IQR 1.9–6 years). The observed 1, 3, and 5‐year median survival rates were 88%, 78%, and 71%, respectively. Thirty‐six patients (26.7%) died during follow‐up with a total of 34 deaths(95%) from a known cause: 27 deaths related to PH complications (81%) and 2 deaths (5.5%) from other causes.

**Figure 1:**
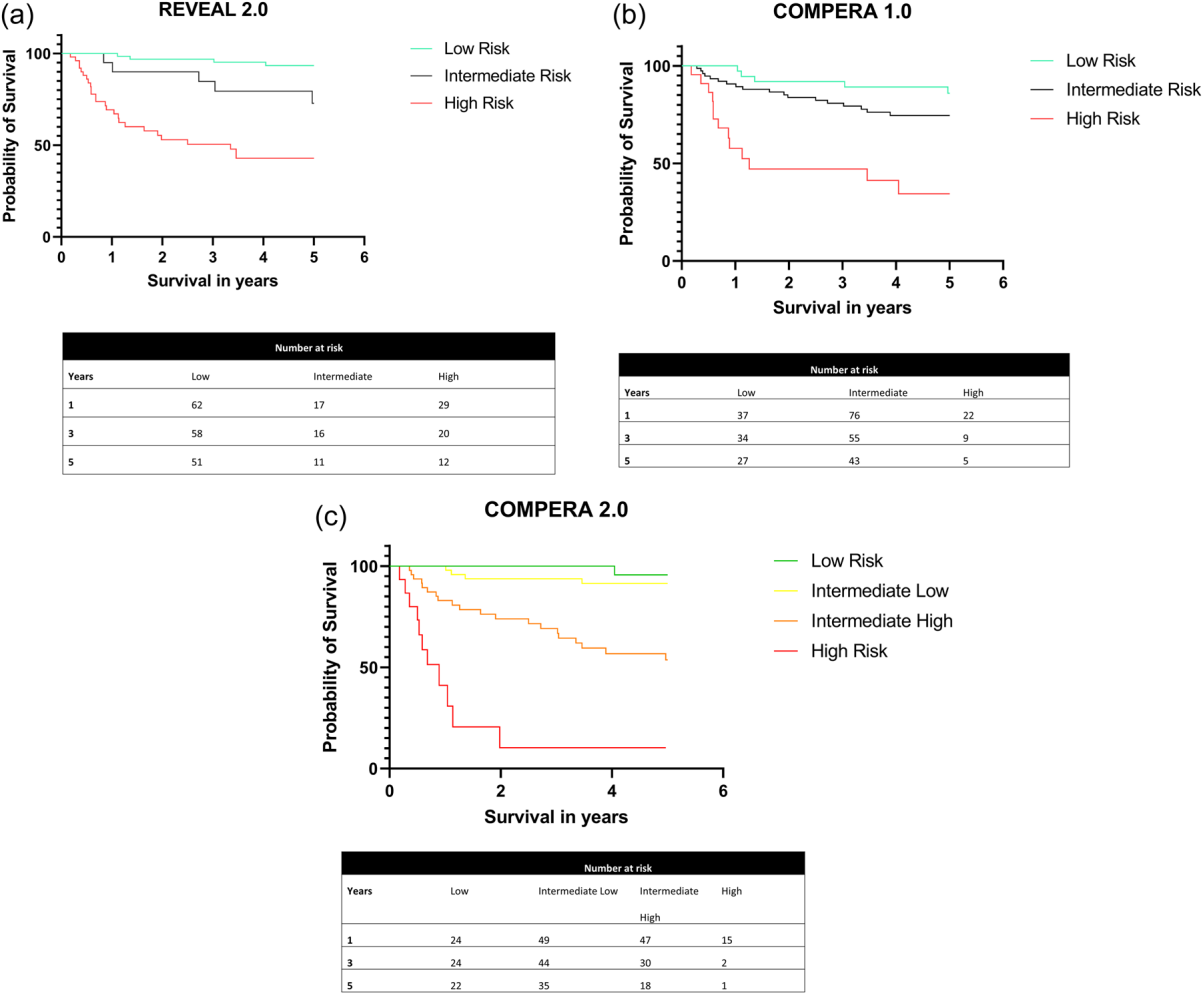
(a) Kaplan–Meier survival estimate of survival probability of pulmonary arterial hypertension patients over 5 years using Registry to Evaluate Early and Long‐term PAH Disease Management (REVEAL 2.0) scoring risk assessment. (b) Kaplan–Meier survival estimate of pulmonary arterial hypertension patients over 5 years using Comparative, Prospective Registry of Newly Initiated Therapies for Pulmonary (COMPERA) scoring risk assessment. (c) Kaplan–Meier survival estimate of pulmonary arterial hypertension patients over 5 years using Comparative, Prospective Registry of Newly Initiated Therapies for Pulmonary (COMPERA) 2.0 scoring risk assessment.

**Figure 2:**
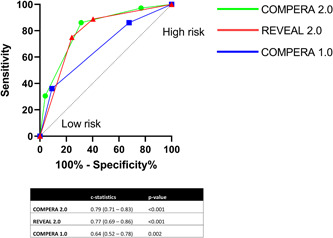
Receiver operating characteristics and corresponding c‐statistics for all three risk scores*. *Three scores include: Registry to Evaluate Early and Long‐term PAH Disease Management 2.0 (REVEAL 2.0), Comparative, Prospective Registry of Newly Initiated Therapies for Pulmonary (COMPERA) 1.0 and COMPERA 2.0. ROC curves and c‐statistics at 3 years after initial visit for the three socres with c‐statistics (95% confidence intervals) and *p* values.

Based on REVEAL 2.0 criteria, 63 (47%) patients were in the low‐risk category, 20 (15%) were at intermediate risk, and 50 (37%) were at high risk. Stratification according to the modified ERS/ESC 3‐strata model Prospective Registry of Newly Initiated Therapies for Pulmonary Hypertension (COMPERA) 1.0 (described in Supporting Information: 2), 37 (27%) were in the low, 76 (56%) intermediate, and 22 (16%) were in the high‐risk category. According to the ESC 4‐strata model COMPERA 2 (Supporting Information: 3), 24 (17.8%), 49 (36.3%), 47 (34.8%), and 15 (11.1%) were in the low, intermediate low, intermediate high‐, and high‐risk categories respectively. According to the REVEAL 2.0 model, 1‐year, 3‐year, and 5‐year survival rates were 98%, 95%, 90% in the low‐risk group, 90%, 79%, 71% in the intermediate‐risk group, and 69, 53, 43 in the high‐risk group, respectively. One‐year, 3‐year, and 5‐year survival in the low COMPERA category was 97, 89, and 85% and 90%, 83, and 74 in the intermediate category and 57, 47, 34% in the high‐risk category, respectively. Stratified according to the ESC 4‐strata model, 1‐year, 3‐year, and 5‐year survival were 100%, 96%, 96% in the low‐risk group, 100%, 93%, 91% in the intermediate low‐risk group, 83%, 69%, 53% in the intermediate high‐risk group; and 41%, 10%, 10% in the high‐risk group, respectively. Kaplan–Meier survival estimates and log‐rank testing showed that all three risk scores provided statistically significant mortality discrimination between the different risk strata (Figure 1a–c). The c‐statistics for REVEAL 2.0 was 0.77 (confidence interval 0.69–0.86, *p* < 0.001), 0.64 (confidence interval 0.52–0.78, *p* = 0.002) for COMPERA 1.0 and 0.79 (confidence interval 0.71–0.83, *p* < 0.001) for COMPERA 2.0, respectively (see Figure 2).

## DISCUSSION

This study aimed to fill a knowledge gap in the outcomes and risk stratification of Hispanic PAH patients, the largest minority group in the United States. This study found that the Hispanic PAH cohort's baseline demographics, functional class, hemodynamics, and PAH‐targeted therapies are comparable to non‐Hispanic PAH cohorts. We found a higher proportion of patients with PAH‐CHD than in other Caucasian‐predominant cohorts. Similar to other PAH cohorts, mortality among Hispanic PAH patients remains high. Using the proposed REVEAL 2.0 and 3‐ or 4‐ category stratification of the modified ESC/ERS models allows for accurate risk prediction for Hispanic PAH patients at baseline. All risk scores seem to apply broadly to Hispanic PAH population, regardless of PAH subgroup.

Even though some differences exist between the REVEAL and the abridged ESC/ERS risk stratifications, both strategies yield excellent prognostic value in largely Caucasian patients with PAH^4^, ^13^, ^16^ A significant limitation in all PAH risk scores developed to date is the lack of data differentiating between ethnic minorities. Although epidemiological data for African‐American patients with PH and PAH are emerging, there is a significant knowledge gap in the risk stratification and prognosis of Hispanic PAH patients. To date, it is unknown if established PAH‐risk predictors are applicable to Hispanic PAH patients. For instance, the REVEAL registry enrolled less than 10% of Hispanic patients, whereas COMPERA did not report race or ethnicity data. The impact of ethnic dissimilarities on cardiovascular disease outcomes is underscored by the fact that despite a similar incidence of CVD risk factors, Hispanics are less likely to develop and die from coronary artery disease^17^ This “Hispanic paradox” indicates that sociodemographic factors, dietary intake, and genetic factors might play a significant role in cardiovascular morbidity, and mortality. Genetic differences certainly determine the pathology and response to therapies in PAH^18^, ^19^ Data on outcomes of Hispanic PAH patients are scarce but do not suggest that Hispanic PAH patients have worse outcomes.^20^, ^21^ In fact, one study applying comprehensive clinical phenotyping and genotyping in different PAH cohorts, found a survival benefit in Hispanic PAH patients, after adjusting for multiple variables.^22^, ^23^ Our study showed a higher 5‐year survival rate of 71%, compared to the REVEAL registry (65%), the Giessen cohort (59%), and the French cohort (67%).^24^, ^25^, ^26^

Several factors may have contributed to this observation. Compared to the REVEAL registry, our cohort consisted only of incident patients, which are prone to survivor bias compared to prevalent cohorts.^27^ Furthermore, the REVEAL registry concluded in 2009 before significant changes in PAH therapeutic approaches.^28^, ^29^, ^30^, ^31^ Upfront combination of PAH‐targeted therapies has demonstrated a reduction in disease progression associated with overall survival benefits in PAH.^23^, ^26^ Sixty‐six percent of patients in this cohort were on upfront combination therapy, compared to 56% in REVEAL and 17% in COMPERA. Secondly, the REVEAL and COMPERA cohorts were published 10 and 5 years before this study was conducted. Over this period, data has emerged showing that upfront combination therapy in intermediate and high‐risk patients is associated with improved functional class and survival. The majority of patients in this cohort were on mono‐therapy and were enrolled between 2015 and 2017. A change in practice patterns with the increasing use of upfront combination therapies may have contributed to the improved survival in this single‐center cohort.

Both the REVEAL 2.0 and the ESC/ERS risk scores, as early as 1 year after treatment and throughout the study period of 5 years, accurately differentiated between patients in our cohort with low, intermediate, and high‐risk for poor outcomes (see Figure 1a–c). Receiver operating characteristics showed that REVEAL 2.0 and COMPERA 2.0 had better sensitivity and specificity to predict 5 year survival, compared to COMEPRA 1.0. In this cohort, the ESC/ERS scores stratified fewer patients in the high‐risk category (3‐strata model 22, 16%, 4‐strata model 15, 11%) compared to the REVEAL 2.0 score (50%, 37%). This difference highlights the inherent bias of both risk tools.^32^ While hemodynamic variables provide a more complete clinical assessment and precise risk stratification, this information is not always available. Furthermore, discrimination by gender, WHO‐group, hospitalization and renal dysfunction are features unique to the REVEAL score and will lead to different proportions of low and high‐risk patients compared to COMPERA. We did not have data on mutation status and no patient in our cohort had an evident family history of hereditary PAH. Compared to the original REVEAL and COMPERA registries, our cohort had a similar proportion of patients with CTD‐PAH (24% vs. 24% in REVEAL and 22% in COMPREA) but a higher proportion of patients with CHD‐PAH (21% vs. 11% in REVEAL and 4% in COMPERA). How these risk scores apply to Hispanic patients with CHD‐PAH is certainly an area that needs further investigation. In the original REVEAL cohort, patients with CHD‐PAH did not seem to have a survival advantage, regardless of the type of defect or repair status.^33^

Another considerable difference between REVEAL and COMPERA is that the former score uses weighted variables based on the contribution of each variable to the overall risk. Variables for the original REVEAL formula were calibrated from a cohort of only 9% Hispanic PAH patients and only 12% CHD‐PAH^33^ Thus, it is possible that the initial risk factor calibration does not translate one‐to‐one in Hispanic PAH patients.

## LIMITATIONS

This study has several limitations, including a relatively small number of patients, single‐center retrospective design, lack of a control group and lack of systematic follow‐up data that would allow REVEAL and ESC risk score assessments after the initiation of PAH‐targeted therapies. Furthermore, our cohort included incident patients only, which are susceptible to survivor bias. To optimize risk prediction, our study was not powered to elucidate which risk factors might carry more weight than others in Hispanic patients.

## CONCLUSION

In summary, our study shows that the REVEAL 2.0, the abridged ESC/ERS 3‐strata, and the 4‐strata risk stratification tools accurately predict outcomes in the treatment of treatment‐nave Hispanic patients at baseline. REVEAL 2.0 can be used to determine a treatment strategy when hemodynamic variables are present. For a noninvasive assessment, the 3‐ and 4‐strate ESC/ERS risk tools provide essential information about the clinical trajectory of an individual patient. Risk assessment in PAH is determined by many nonmodifiable risk factors, such as age, gender, and type of PAH, as well as modifiable factors such as comorbidities and symptoms. Future studies are needed to further understand modifiable risk factors based on ethnicity. This will contribute to a personalized medicine approach for the risk stratification and the management of PAH patients.

## AUTHOR CONTRIBUTIONS

Jose B. Cruz Rodriguez, and Khan O. Mohammad collected data; Kahtan Fadah, and Nils P. Nickel wrote the original manuscript; Debabrata Mukherjee, Haider Alkhateeb, Hernando Garcia, Dan Schuller, and Sandeep Sahay revised the manuscript and provided key feedback and insights. Kahtan Fadah, and Nils P. Nickel analyzed and created figures, tables, and Supporting Information. Nils P. Nickel supervised the project.

## CONFLICTS OF INTEREST STATEMENT

Sandeep Sahay declare he is an advisor for Janssen, Bayer, United Therapeutics, Gossamer Bio, Altavant sciences, MERCK; Speaker United Therapeutics, Janssen; Steering committee member for Bayer and Liquidia technologies; Clinical Trial support from Bayer, Janssen, United Therapeutics, Gossamer Bio, Altavant Sciences, Keros; Received Research Grant from United Therapeutics. The remaining authors declare no conflict of interest.

## ETHICS STATEMENT

Written informed consent was not required for this study and was performed in accordance with Texas Tech University Institutional Review Board.

## Supporting information

Supporting information.

## References

[1] Bartolome S , Hoeper MM , Klepetko W . Advanced pulmonary arterial hypertension: mechanical support and lung transplantation. Eur Respir Rev. 2017;26. 10.1183/16000617.0089-2017 PMC9488526 29263172

[2] Mannino DM , Thomashow B . Reducing COPD readmissions great promise but big problems. Chest. 2015;147:1199–201. 25940241 10.1378/chest.15-0380

[3] D'Alonzo GE , Barst RJ , Ayres SM , Bergofsky EH , Brundage BH , Detre KM , Fishman AP , Goldring RM , Groves BM , Kernis JT . Survival in patients with primary pulmonary hypertension: results from a national prospective registry. Ann Intern Med. 1991;115(5):343–9. 1863023 10.7326/0003-4819-115-5-343

[4] Gong SG , Wu WH , Li C , Zhao Q‐H , Jiang R , Luo C‐J , Qio H‐L , Liu J‐M , Wang L , Zhang R . Validity of the ESC risk assessment in idiopathic pulmonary arterial hypertension in China. Front Cardiovasc Med. 2021;8:1663. 10.3389/fcvm.2021.745578 PMC8645595 34881304

[5] Ling Y , Johnson MK , Kiely DG , Condliffe R , Elliot CA , Gibbs JSR , Howard LS , Pepke‐Zaba J , Sheares KKK , Corris PA , Fisher AJ , Lordan JL , Gaine S , Coghlan JG , Wort SJ , Gatzoulis MA , Peacock AJ . Changing demographics, epidemiology, and survival of incident pulmonary arterial hypertension: results from the pulmonary hypertension registry of the United Kingdom and Ireland. Am J Respir Crit Care Med. 2012;186:790–6. 22798320 10.1164/rccm.201203-0383OC

[6] Ventetuolo CE , Praestgaard A , Palevsky HI , Klinger JR , Halpern SD , Kawut SM . Sex and haemodynamics in pulmonary arterial hypertension. Eur Respir J. 2014;43:523–30. 23949961 10.1183/09031936.00027613 PMC4338984

[7] Gall H , Felix JF , Schneck FK , Milger K , Sommer N , Voswinckel R , Franco OH , Hofman A , Schermuly RT , Weissmann N , Grimminger F , Seeger W , Ghofrani HA . The Giessen pulmonary hypertension registry: survival in pulmonary hypertension subgroups. J Heart Lung Transplant. 2017;36:957–67. 28302503 10.1016/j.healun.2017.02.016

[8] Hoeper MM , Pausch C , Grünig E , Klose H , Staehler G , Huscher D , Pittrow D , Olsson KM , Vizza CD , Gall H , Benjamin N , Distler O , Opitz C , Gibbs JSR , Delcroix M , Ghofrani HA , Rosenkranz S , Ewert R , Kaemmerer H , Lange TJ , Kabitz HJ , Skowasch D , Skride A , Jureviciene E , Paleviciute E , Miliauskas S , Claussen M , Behr J , Milger K , Halank M , Wilkens H , Wirtz H , Pfeuffer‐Jovic E , Harbaum L , Scholtz W , Dumitrescu D , Bruch L , Coghlan G , Neurohr C , Tsangaris I , Gorenflo M , Scelsi L , Vonk‐Noordegraaf A , Ulrich S , Held M . Idiopathic pulmonary arterial hypertension phenotypes determined by cluster analysis from the COMPERA registry. J Heart Lung Transplant. 2020;39:1435–44. 33082079 10.1016/j.healun.2020.09.011

[9] Mukherjee D . ESC/ERS guidelines for pulmonary hypertension: key points—American College of Cardiology 2022 Aug 30; latest‐in‐cardiology (ten‐points‐to‐remember). [Internet] [cited 2022 Sep 24]. 2022. Available from: https://www.acc.org/Latest-in-Cardiology/ten-points-to-remember/2022/08/30/19/11/2022-ESC-Guidelines-for-Pulmonary-Hypertension-ESC-2022

[10] Humbert M , Kovacs G , Hoeper MM , Badagliacca R , Berger RMF , Brida M , Carlsen J , Coats AJS , Escribano‐Subias P , Ferrari P , Ferreira DS , Ghofrani HA , Giannakoulas G , Kiely DG , Mayer E , Meszaros G , Nagavci B , Olsson KM , Pepke‐Zaba J , Quint JK , Radegran G , Simonneau G , Sitbon O , Tonia T , Toshner M , Vachiery JL , Noordegraaf, AV , Delcroix M , Rosenkranz S , Schwerzmann M , Dinh‐Xuan A‐T , Bush A , Abdelhamid M , Aboyans V , Arbustini E , Asteggiano R , Barbera J‐A , Beghetti M , Cikes M , Condliffe R , de Man F , Falk V , Fauchier L , Gaine S , Galie N , Gin‐Sing W , Granton J , Grunig E , Hassoun PM , Hellemons M . 2022 ESC/ERS guidelines for the diagnosis and treatment of pulmonary hypertension developed by the task force for the diagnosis and treatment of pulmonary hypertension of the European Society of Cardiology (ESC) and the European Respiratory Society (ERS). Endorsed by the International Society for Heart and Lung Transplantation (ISHLT) and the European Reference Network on rare respiratory diseases (ERN‐LUNG). Eur Heart J [Internet]. 2022;43:ehac237. https://research.rug.nl/en/publications/2022-escers-guidelines-for-the-diagnosis-and-treatment-of-pulmona

[11] Boucly A , Weatherald J , Savale L , Jaïs X , Cottin V , Prevot G , Picard F , de Groote P , Jevnikar M , Bergot E , Chaouat A , Chabanne C , Bourdin A , Parent F , Montani D , Simonneau G , Humbert M , Sitbon O . Risk assessment, prognosis and guideline implementation in pulmonary arterial hypertension. Eur Respir J. 2017;50:1700889. 28775050 10.1183/13993003.00889-2017

[12] Hoeper MM , Kramer T , Pan Z , Eichstaedt CA , Spiesshoefer J , Benjamin N , Olsson KM , Meyer K , Vizza CD , Vonk‐Noordegraaf A , Distler O , Opitz C , Gibbs JSR , Delcroix M , Ghofrani HA , Huscher D , Pittrow D , Rosenkranz S , Grünig E . Mortality in pulmonary arterial hypertension: prediction by the 2015 European pulmonary hypertension guidelines risk stratification model. Eur Respir J. 2017;50(2):1700740. 28775047 10.1183/13993003.00740-2017

[13] Benza RL , Gomberg‐Maitland M , Elliott CG , Farber HW , Foreman AJ , Frost AE , McGoon MD , Pasta DJ , Selej M , Burger CD , Frantz RP . Predicting survival in patients with pulmonary arterial hypertension. Chest. 2019;156:323–37. 30772387 10.1016/j.chest.2019.02.004

[14] Heidenreich PA , Bozkurt B , Aguilar D , Allen LA , Byun J‐J , Colvin MM , Deswal A , Drazner MH , Dunlay SM , Evers LR , Fang JC , Fedson SE , Fonarow GC , Hayek SS , Hernandez AF , Khazanie P , Kittleson MM , Lee CS , Link MS , Milano CA , Nnacheta LC , Sandhu AT , Stevenson LW , Vardeny O , Vest AR , Yancy CW . American College of Cardiology/American Heart Association/Heart Failure Society of America Guideline for the management of heart failure: executive summary. J Card Fail. 2022;79:17. 10.1016/j.cardfail.2022.02.009 35378259

[15] Unger T , Borghi C , Charchar F , Khan NA , Poulter NR , Prabhakaran D , Ramirez A , Schlaich M , Stergiou GS , Tomaszewski M , Wainford RD , Williams B , Schutte AE . 2020 international society of hypertension global hypertension practice guidelines. Hypertension. 2020;75(6):1334–57. https://www.ahajournals.org/doi/abs/10.1161/HYPERTENSIONAHA.120.15026 32370572 10.1161/HYPERTENSIONAHA.120.15026

[16] Anderson JJ , Lau EM , Lavender M , Benza R , Celermajer DS , Collins N , Corrigan C , Dwyer N , Feenstra J , Horrigan M , Keating D , Kermeen F , Kotlyar E , McWilliams T , Rhodes B , Steele P , Thakkar V , Williams T , Whitford H , Whyte K , Weintraub R , Wrobel JP , Keogh A , Strange G . Retrospective validation of the REVEAL 2.0 risk score with The Australian and New Zealand pulmonary hypertension registry cohort. Chest. 2020;157(1):162–72. http://journal.chestnet.org/article/S0012369219339340/fulltext 31563497 10.1016/j.chest.2019.08.2203

[17] Medina‐Inojosa J , Jean N , Cortes‐Bergoderi M , Lopez‐Jimenez F . The Hispanic paradox in cardiovascular disease and total mortality. Prog Cardiovasc Dis. 2014;57:286–92. 25246267 10.1016/j.pcad.2014.09.001

[18] Gabler NB , French B , Strom BL , Liu Z , Palevsky HI , Taichman DB , Kawut SM , Halpern SD . Race and sex differences in response to endothelin receptor antagonists for pulmonary arterial hypertension. Chest. 2012;141:20–6. 21940766 10.1378/chest.11-0404 PMC5991545

[19] Mata‐Greenwood E , Chen DB . Racial differences in nitric oxide‐dependent vasorelaxation. Reprod Sci. 2008;15:9–25. 18212350 10.1177/1933719107312160 PMC2459254

[20] Hispanic ethnicity and social determinants of health in pulmonary arterial hypertension: the pulmonary hypertension association registry. Ann Am Thorac Soc. 19:(9). 10.1513/AnnalsATS.202109-1051OC PMC12039925 35239467

[21] Farber HW , Miller DP , Poms AD , Badesch DB , Frost AE , Rouzic EML , Romero AJ , Benton WW , Elliott CG , McGoon MD , Benza RL . Five‐year outcomes of patients enrolled in the REVEAL registry. Chest. 2015;148:1043–54. 26066077 10.1378/chest.15-0300

[22] Karnes JH , Wiener HW , Schwantes‐An TH , Natarajan B , Sweatt AJ , Chaturvedi A , Arora A , Batai K , Nair V , Steiner HE , Giles JB , Yu J , Hosseini M , Pauciulo MW , Lutz KA , Coleman AW , Feldman J , Vanderpool R , Tang H , Garcia JGN , Yuan JXJ , Kittles R , de Jesus Perez V , Zamanian RT , Rischard F , Tiwari HK , Nichols WC , Benza RL , Desai AA . Genetic admixture and survival in diverse populations with pulmonary arterial hypertension. Am J Respir Crit Care Med. 2020;201(11):1407–15. 31916850 10.1164/rccm.201907-1447OC PMC7258627

[23] Karnes JH , Wiener HW , Schwantes‐An TH , Natarajan B , Sweatt AJ , Chaturvedi A , Arora A , Batai K , Nair V , Steiner HE , Giles JB , Yu J , Hosseini M , Pauciulo MW , Lutz KA , Coleman AW , Feldman J , Vanderpool R , Tang H , Garcia JGN , Yuan JXJ , Kittles R , de Jesus Perez V , Zamanian RT , Rischard F , Tiwari HK , Nichols WC , Benza RL , Desai AA . Genetic admixture and survival in diverse populations with pulmonary arterial hypertension. Am J Respir Crit Care Med. 2020;201(11):1407–15. 31916850 10.1164/rccm.201907-1447OC PMC7258627

[24] Humbert M , Sitbon O , Yaici A , Montani D , O'Callaghan DS , Jais X , Parent F , Savale L , Natali D , Gunther S , Chaouat A , Chabot F , Cordier JF , Habib G , Gressin V , Jing ZC , Souza R , Simonneau G . Survival in incident and prevalent cohorts of patients with pulmonary arterial hypertension. Eur Respir J. 2010 Sep 1;36(3):549–55. 20562126 10.1183/09031936.00057010

[25] Gall H , Felix JF , Schneck FK , Milger K , Sommer N , Voswinckel R , Franco OH , Hofman A , Schermuly RT , Weissmann N , Grimminger F , Seeger W , Ghofrani HA . The giessen pulmonary hypertension registry: survival in pulmonary hypertension subgroups. J Heart Lung Transplant. 2017 Sep 1;36(9):957–67. 28302503 10.1016/j.healun.2017.02.016

[26] Mannino DM , Thomashow B . Reducing COPD readmissions great promise but big problems. Chest. 2015;147:1199–201. 25940241 10.1378/chest.15-0380

[27] Miller DP , Gomberg‐Maitland M , Humbert M . Survivor bias and risk assessment. Eur Respir J. 2012;40(3):530–2. https://erj.ersjournals.com/content/40/3/530 22941543 10.1183/09031936.00094112

[28] Boucly A , Savale L , Jaïs X , Bauer F , Bergot E , Bertoletti L , Beurnier A , Bourdin A , Bouvaist H , Bulifon S , Chabanne C , Chaouat A , Cottin V , Dauphin C , Degano B , De Groote P , Favrolt N , Feng Y , Horeau‐Langlard D , Jevnikar M , Jutant EM , Liang Z , Magro P , Mauran P , Moceri P , Mornex JF , Palat S , Parent F , Picard F , Pichon J , Poubeau P , Prévot G , Renard S , Reynaud‐Gaubert M , Riou M , Roblot P , Sanchez O , Seferian A , Tromeur C , Weatherald J , Simonneau G , Montani D , Humbert M , Sitbon O . Association between initial treatment strategy and long‐term survival in pulmonary arterial hypertension. Am J Respir Crit Care Med. 2021;204:842–54. 34185620 10.1164/rccm.202009-3698OC

[29] Chang KY , Duval S , Badesch DB , Bull TM , Chakinala MM , Marco TD , Frantz RP , Hemnes A , Mathai SC , Rosenzweig EB , Ryan JJ , Thenappan T . Mortality in pulmonary arterial hypertension in the modern era: early insights from the pulmonary hypertension association registry. J Am Heart Assoc. 2022;11:e024969. 35475351 10.1161/JAHA.121.024969 PMC9238604

[30] Studer S , Hull M , Pruett J , Elliott C , Tsang Y , Drake W . Retrospective database analysis of treatment patterns among patients with pulmonary arterial hypertension. Pulm Ther. 2020;6:79–92. 32048240 10.1007/s41030-019-00106-4 PMC7229082

[31] Chin KM , Sitbon O , Doelberg M , Feldman J , Gibbs JSR , Grünig E , Hoeper MM , Martin N , Mathai SC , McLaughlin VV , Perchenet L , Poch D , Saggar R , Simonneau G , Galiè N . Three‐ versus two‐drug therapy for patients with newly diagnosed pulmonary arterial hypertension. JACC. 2021;78:1393–403. 34593120 10.1016/j.jacc.2021.07.057

[32] Miller DP , Gomberg‐Maitland M , Humbert M . Survivor bias and risk assessment. Eur Respir J. 2012;40:530–2. 22941543 10.1183/09031936.00094112

[33] Benza RL , Miller DP , Gomberg‐Maitland M , Frantz RP , Foreman AJ , Coffey CS , Frost A , Barst RJ , Badesch DB , Elliott CG , Liou TG , McGoon MD . Predicting survival in pulmonary arterial hypertension: insights from the registry to evaluate early and long‐term pulmonary arterial hypertension disease management (REVEAL). Circulation. 2010;122:164–72. 20585012 10.1161/CIRCULATIONAHA.109.898122

