# Supplementary material for: Prognosis in Hispanic patient population with pulmonary arterial hypertension: An application of common risk stratification models
Source: Pulm Circ. 2023 Apr 1;13(2):e12209. doi: 10.1002/pul2.12209 (PMC10069240; doi:10.1002/pul2.12209)
Supplement: Supplementary file 1 — Supporting information. [file PUL2-13-e12209-s001.docx]

Supplement 1. Classification system according to the Registry to Evaluate Early and Long-term PAH Disease Management (REVEAL 2.0) .


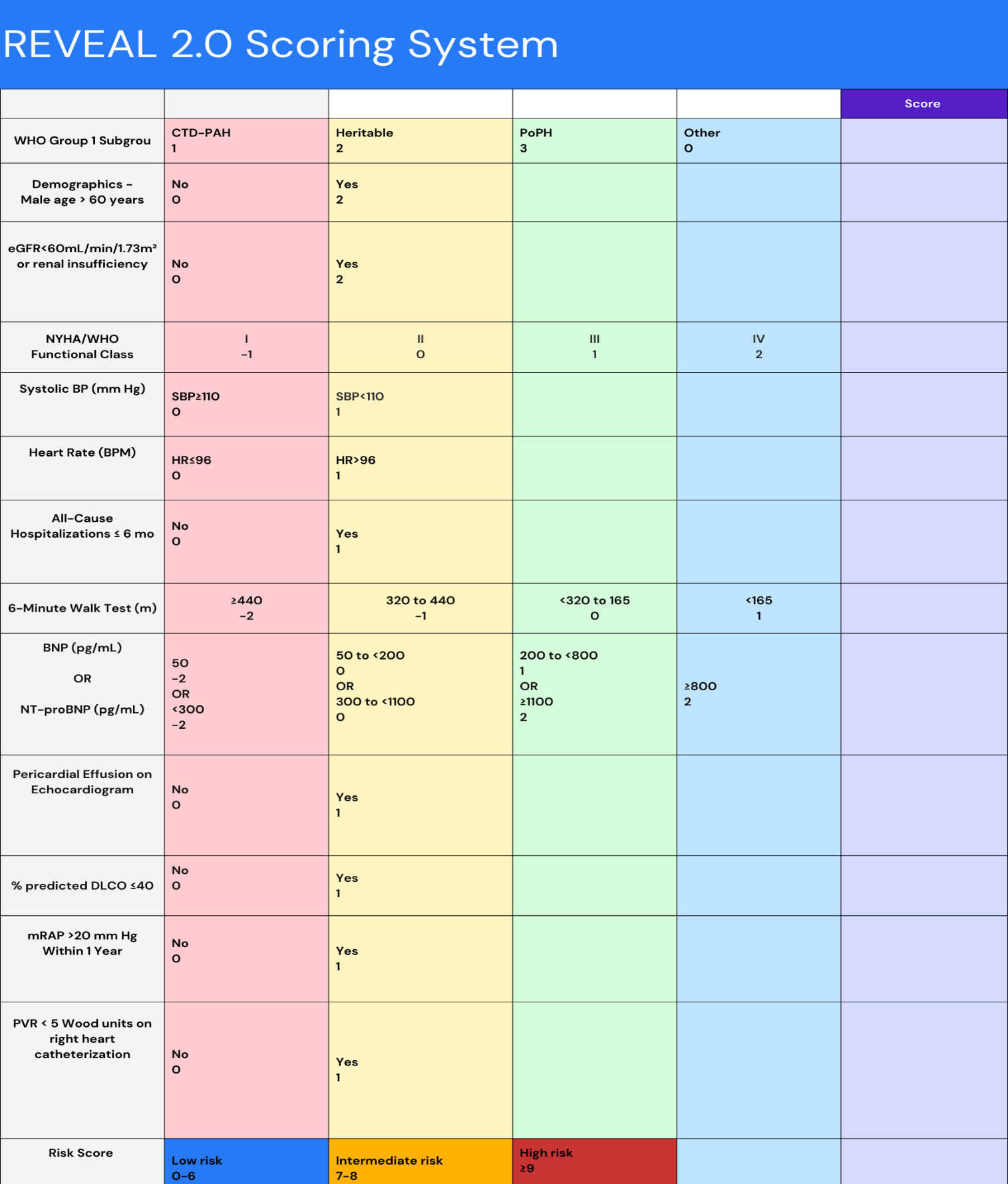


Patients were classified into low-, intermediate- and high-risk groups based on World Health Organization (WHO)-group, and the following variables: demographics, renal function, functional class, 6-minute walk testing, vital signs, brain natriuretic peptide (BNP)/N-terminal prohormone of brain natriuretic peptide (NT-proBNP), presence of a pericardial effusion on echocardiogram, diffusing capacity for carbon monoxide (DLCO) on pulmonary function testing, mean right arterial pressure, and pulmonary vascular resistance (13). The score included weighted variables based on an integer score for a risk factor in proportion to its contribution to the overall risk rating.

Supplement 2: Shows Criteria for Stratification according to the modified three- and four-stratum model; Prospective Registry of Newly Initiated Therapies for Pulmonary Hypertension (COMPERA) 2.0


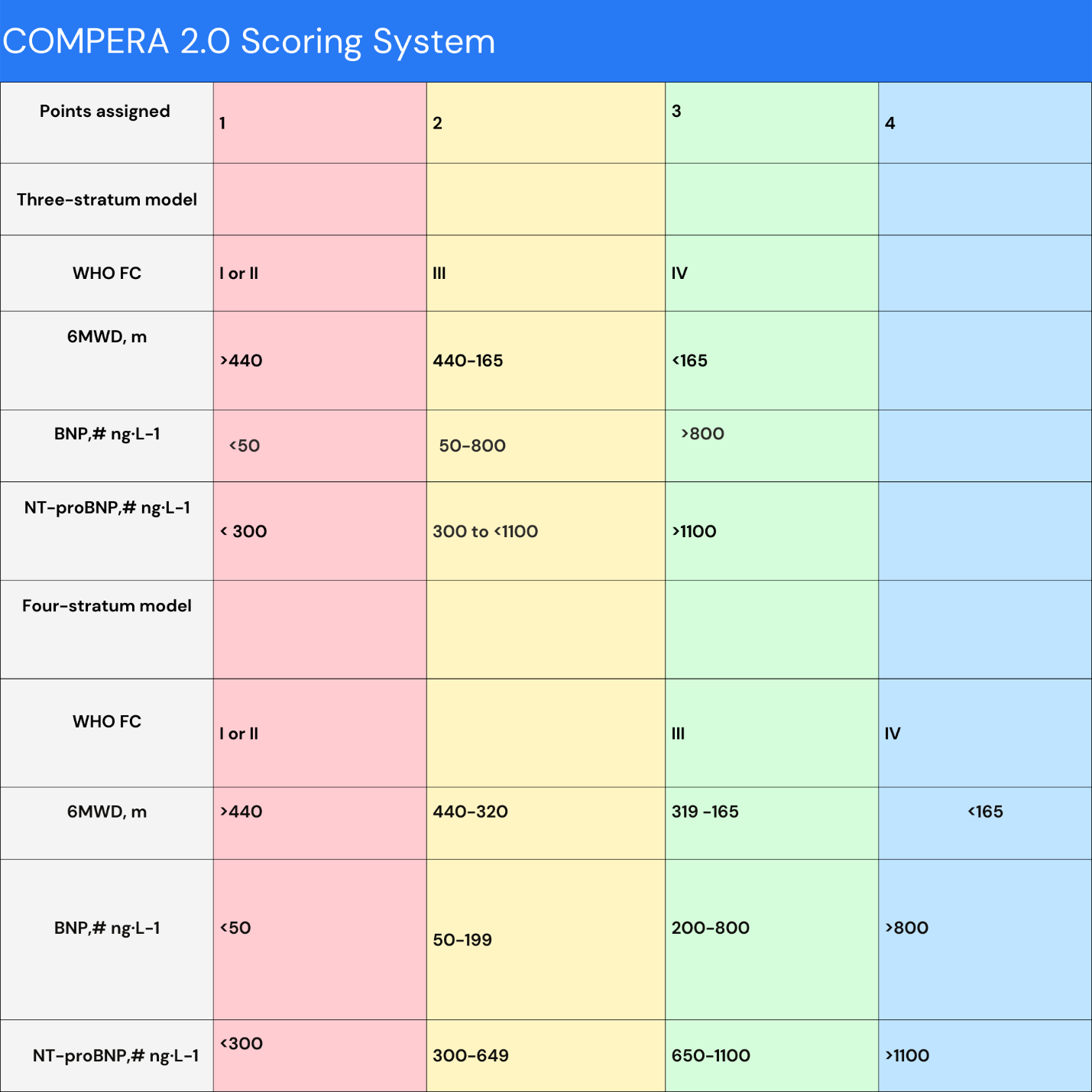


# Refers to the number of cut-off values for all non-invasive variables used for COMPERA 2.0: 6-minute walk testing (6MWD), brain natriuretic peptide (BNP)/N-terminal prohormone of brain natriuretic peptide (NT-proBNP), and World Health Organization Functional Class (WHO-FC) with points system.

Supplement 3: Criteria for Stratification according to the Comparative, Prospective Registry of Newly Initiated Therapies for Pulmonary (COMPERA) 2.0


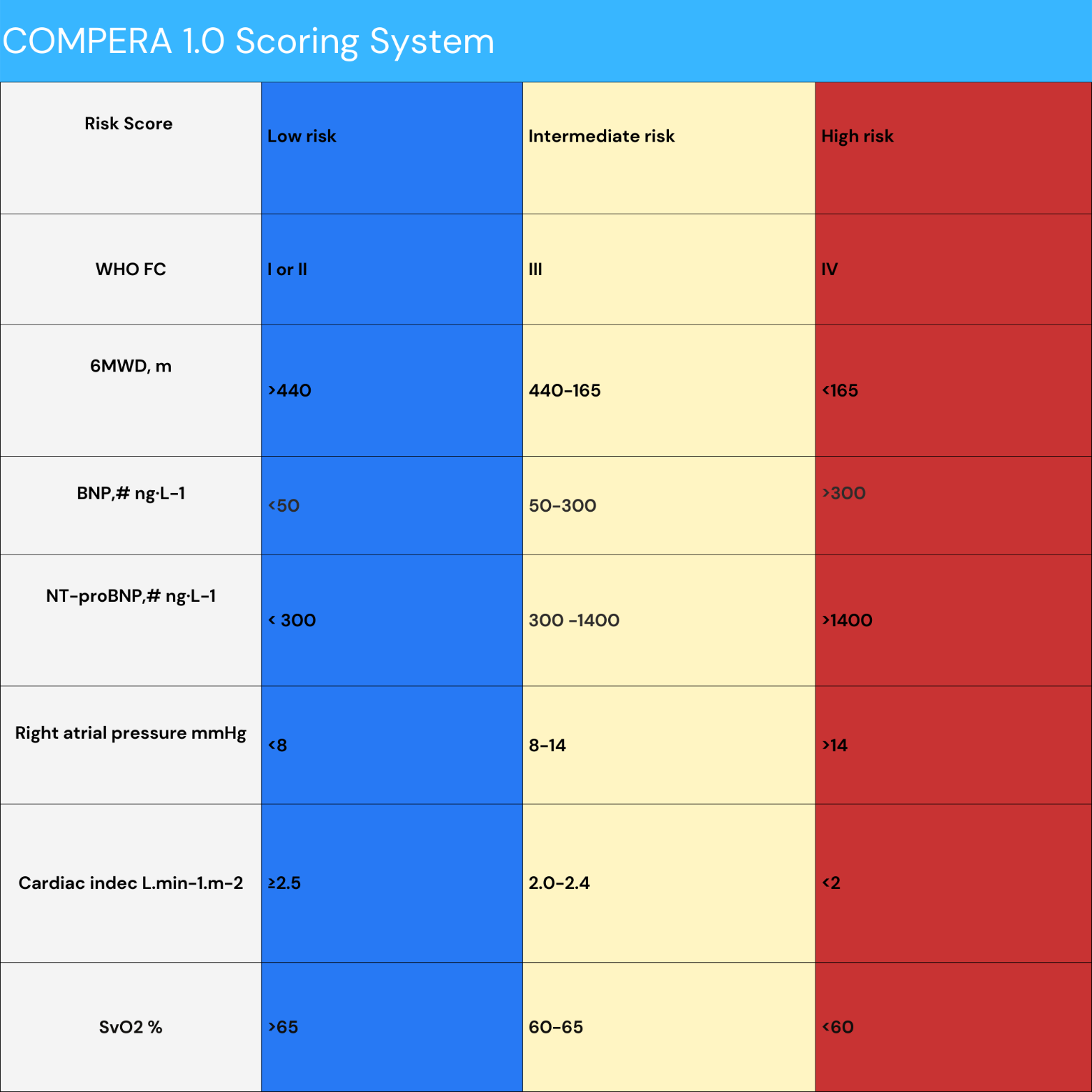
Risk score used to determine low, intermediate or high risk patients according World Health Organization Functional Class (WHO-FC), six-minute walking distance (6mwd), NT-pro-B-type Natriuretic Peptide (NT-proBNP), right arterial pressure, cardiac index, Mixed venous oxygen saturation (SvO2).
